# GNPs-CS/KGM as Hemostatic First Aid Wound Dressing with Antibiotic Effect: In Vitro and In Vivo Study

**DOI:** 10.1371/journal.pone.0066890

**Published:** 2013-07-16

**Authors:** Li Fan, Chong Cheng, Youbei Qiao, Fei Li, Wei Li, Hong Wu, Bo Ren

**Affiliations:** 1 Department of Pharmaceutical Chemistry and Analysis, School of Pharmacy, Fourth Military Medical University, Xi'an, Shaanxi, China; 2 Department of Anesthesia, 302 Military Hospital of China, Beijing, China; Dowling College, United States of America

## Abstract

Ideal wound dressing materials should create a good healing environment, with immediate hemostatic effects and antimicrobial activity. In this study, chitosan/konjac glucomannan (CS/KGM) films embedded with gentamicin-loaded poly(dex-GMA/AAc) nanoparticles (giving GNP-CS/KGM films) were prepared as novel wound dressings. The results revealed that the modified CS/KGM films could be used as effective wound dressings and had significant hemostatic effects. With their microporous structure, the films could effectively absorb water from blood and trap blood cells. The gentamicinloaded poly(dex-GMA/AAc) nanoparticles (GNPs) also further promoted blood clotting, with their favorable water uptake capacity. Thus, the GNP-CS/KGM films had wound healing and synergistic effects that helped to stop bleeding from injuries, and also showed good antibiotic abilities by addition of gentamicin to the NPs. These GNPCS/KGM films can be considered as promising novel biodegradable and biocompatible wound dressings with hemostatic capabilities and antibiotic effects for treatment of external bleeding injuries.

## Introduction

Ideal wound dressing materials should have following characteristics [1]–[3]: create a moist wound healing environment, absorb excess exudate, allow gaseous exchange, be removed easily without trauma to the wound and better with effects of hemorrhage control, antimicrobial activities, nontoxic and biocompatible.

Many efforts have been made to prepare such wound healing materials including proteins (e.g., fibrinogen [4], thrombin [5], collagen [6], gelatin [7], albumin [8]), and polysaccharides [9] (chitosan, chitin, poly (Nacetyl glucosamine) and cellulose). Although most of these materials have been proved valuable for wound healing [10]–[13] and hemorrhage control [14]–[16] in many cases, their main limitations are lack of efficacy in bleeding, anti-inflammatory and antibiosis.

In recent years, scientists have attempted to apply dehydrated zeolite material to the bleeding site in order to induce hemostasis through dehydration of the wounded area. However, zeolite particles possess poor adhesive properties, and they will release heat when in contact with water [17]. Many biomaterials based nanoparticles, such as modified polysaccharides with acrylic polymers [18]–[20], with similar strong capabilities of water uptake with zeolite particles, could absorb large amount water from blood to form a clot quickly. And polysaccharides dressing itself, such as chitosan (CS) dressing, possessing blood clot formation activity and cell adhesiveness [21] has already been proved to have desirable qualities for wound h ealing [22] and hemostasis [23]. Thus, we hypothesized that if CS dressings composited with polysaccharides based nanoparticles may have great valuable for wound healing and hemorrhage control.

However, CS dressing has poor tensile strength and elasticity. Hence development of high strength composites that are biocompatible and that can help in wound healing may be necessary for CS as wound dressing applications. Improvement of water absorbing and water permeating properties is also necessary for the products derived from CS. Konjac glucomannan (KGM), could significantly improve mechanical properties of CS as a wound dressing materials. The tensile strength and breaking elongation of blend films were enhanced about 40% and 30%, respectively [24]. Moreover, the addition of KGM improves the biocompatibility of CS materials [25].

Though films based on KGM/CS has been found to be nontoxic, biodegradable, biofunctional, biocompatible in addition to having antimicrobial characteristics [26], [27], the antibacterial activity of chitosan was inferior to that of the organic antibacterial compounds and could not provide efficient antimicrobial activity or a continuous and sustained release of the antibacterial agent on the wound surface.

In recent times, there has been considerable interest in preparations of antibiotics loaded nanoparticles and films in order to enhance the antimicrobial activity of wound dressing [28]. It was reported by R. Hamblin that a dressing combining CS acetate with silver nanoparticles leaded to improved antimicrobial efficacy against fatal infections [28]. In our previous study, we have developed nanoparticles based on derivative dextran that have shown great capabilities in drug-controlled release [29], [30].

In this study, poly (dex-GMA/AAc) nanoparticles were also used as antibiotics gentamicin delivery vehicles in order to keep gentamicin sustainable release. We kept on adjusting ratio between KGM and CS in order to obtain more efficient wound dressing film with better tensile strength and breaking elongation. It was revealed by research result that gentamicin got well sustainable drug release profile from poly (dex-GMA/AAc) nanoparticles. And the antibacterial test result revealed that it possessed continuously bacteriostatic activity after adhere to skin surface. Also, it was confirmed by in vitro and vivo study that CS/KGM film was valuable for wound healing and hemorrhage control due to its significant promoting wound healing effect and fast hemostatic effect.

## Materials and Methods

### Materials

Dextran (Mn∼70,000 g/mol) was obtained from Leuconostoc spp., N, N-Dimethylpyridin-4-amine (DMAP, 99%), Glycidyl methacrylate (GMA, 97%), Chitosan (Mn∼75,000 g/mol, 75–85% deacetylated), and Gentamicin were purchased from Sigma-Aldrich. Dimethylsulfoxide (DMSO), N, N'-Methylenebisacrylamide (MBA), ammonium persulfate (APS), acrylic acid (AA), acetylacetone, and other chemical agent were acquired from Fluka. Konjac Glucomannan (KGM) from Chengdu new interstate development Co., LTD, Dulbecco's modified Eagle media (DMEM) from Gibco and fetal calf serum (FBS) were used without further purification. Phosphate buffered saline (PBS) was prepared by dissolving 8.00 g NaCl, 0.20 g KCl, 1.15 g Na_2_HPO_4_, and 0.24 g KH_2_PO_4_ into ∼900 mL of water. The pH was adjusted to 7.40 with 1 M NaOH or 1 M HCl, and the solution was mixed with additional water to 1.00 L in a volumetric flask. Bacteria strains staphylococcus aureus (ATCC 25923), escherichia coli (ATCC 25922) and Pseudomonas aeruginosa (ATCC 27853) were obtained from Dept. of Laboratory in Xijing hospital. Yunnan baiyao as a positive control was also obtained from Xijing hospital.

### Poly (DEX-GMA/AAc) blank nanoparticles and Gentamicin loaded nanoparticles synthesis and characterization

**Figure 1:**
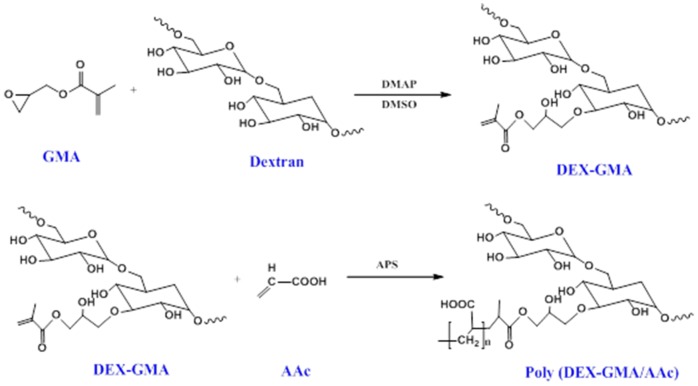
Schematic of derivatization of dextran and the free-radical mediated polymerization of the crosslinked polymer nanoparticles.

DEX-GMA precursor and Poly (DEX-GMA/AAc) nanoparticles were synthesized as has been previously reported [30] in our paper. Though 3 methods have been reported in our previous paper for synthesis of Poly (DEX-GMA/AAc) nanoparticles, method of free radical polymerization was testified to be the preferred one with best repeatability and size distribution (As shown in Figure 1). In brief, dextran (5.0 g) and DMAP (1.0 g) was dissolved in 50 ml of DMSO at room temperature. After dissolution of DMAP, GMA (0.8 g) was added. The mixture was stirred for 30 h at room temperature under nitrogen. The obtain dextran polymer was then precipitated with ethanol and fluffy product polymers were obtained. The polymers were further dissolved in deionized water and reprecipitated out with ethanol three times. The product was dispersed into distilled water, dialyzed for 1 week at 4°C. After lyophilizing, the white Dex-GMA was obtained. The purified Dex-GMA was characterized by ^1^H-NMR spectroscopy. Poly (DEX-GMA/AAc) blank nanoparticles were synthesized in 30 ml pH 8.0 phosphate buffers by a free radical emulsion polymerization. Gentamicin loaded nanoparticles were obtained as the same method with initially adding Gentamicin (50 mg). AAc (0.2 g) was dissolved in 5 mL PBS and then neutralized by NaOH solution (0.25 mol/L). Dex-GMA (0.6 g) and MBA (2 mg/mL, 15 mL) were added into AAc solution and obtained mixture 1. Tween-80 (0.1 mL) as emulsifier was added directly into PBS, and mixture 1 was dropwised added into the solution. The reaction mixture was purged with nitrogen for 20 min and the reaction temperature was increased up to 70°C. APS (10% w/v, 1 mL) as initiator was added and reacted for 5 h under nitrogen. Poly (dex-GMA/AAc) nanoparticles were collected by centrifugation at 12000 rpm for 30 min. Excess surfactant and unencapsulated gentamicin were removed by dialysis (dialysis bag with 10000 MWCO) for 1 day and then nanoparticles solution was lyophilized. The blank and drug loaded nanoparticles were characterized for their size and surface morphology by dynamic laser scattering (DLS) (Malvern Zetasizer Nano S90) and transmission electron microscopy (TEM) (Hitachi HT-7700).

Gentamicin encapsulation efficiency (EE) and loading efficiency (LE) were determined by dissolving 100 mg of drug loaded nanoparticles in 50 ml PBS buffer with 5 ml 0.1 mol/l HCl for 12 h under 90°C water bath. Then filter the solution using Millipore Ultrafiltration (UF) membranes with MWCO 1000 and the filtrate was brought to volume of 100 mL. Gentamicin was diluted with 5 ml of water by vortexing and assayed photometrically (310 nm) after derivation with *o*-phthalaldehyde [31]. EE and LE were calculated by the formula below
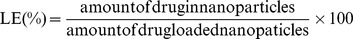


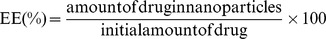



### KGM/CS film preparation and characterization

KGM/CS membrane was prepared following Zhang's previous paper [32] using casting and solvent evaporation technique [33], [34] with some modification. KGM was purified by extraction of phenol and ethanol (4∶1, v/v) for 5 times and extraction of chloroform and ethanol (5∶1, v/v) for 3 times. Purified KGM was obtained after vacuum dried. Then purified totally soluble KGM was dissolved in distilled water to a concentration of 1 wt%. CS was dissolved in a 1wt% aqueous acetic acid to prepare a concentration of 1 wt% solution. The solutions of KGM and CS with different mixing ratios [25/75, 50/50, and 75/25 KGM/CS (w/w)] were cast onto polystyrene plates and lyophilized. A series of blend membranes were there by obtained and coded as K25C75, K50C50, and K75C25. Pure CS film and pure KGM film were prepared following the same protocol. The films were seal up for safekeeping after Co-60 irradiation. Film samples of about 100 μm thickness were coated with gold in 0.1Torr (0.13 mBar ) vacuum degrees. The cross section morphologies were observed on a Hitachi S-3400N SEM. The tensile strength (TS) and breaking elongation (E) of the films were measured on an Instron apparatus (Model 3342, Instron Corp., Canton, MA). The film (0.2×0.3 cm) were used in the stretch test under the stress of 1 and 2 N. Draft distance of clip was set as 20 mm with stretching speed of 50 mm·min^−1^. TS and E were calculated following the formula below
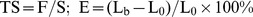
F _(N)_: maximum stress of stretching, S _(mm_^2^_)_: initial sectional area of sample; L_0_: initial sample length; L_b_: sample length at breakage (n = 6).

The swelling behaviors of pure CS and CS/KGM blend films were studied according to the method described by Wang et al. [35]. The samples of a cylinder shape (diameter 30 mm, thickness 4 mm) were weighted (W_o_) before immersing in PBS at 37°C and immersed in PBS for different time intervals and weighted (Ws). The swelling degree in PBS (SDP) was calculated using the following equation:
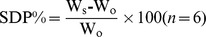
Where Ws is the weight of the hydrogel swollen in PBS, Wo is the initial weight of hydrogel samples. The results are presented as a mean value with a standard deviation (n = 6).

The water vapor transmission rate (WVTR) across the pure CS and CS/KGM blend films was determined to the method described by Tsao et al. [36]. The pure CS and CS/KGM blend films were cut into 20 mm×20 mm with a thickness of 3 mm, and mounted on the mouth of cylindrical aluminium cups (14 mm diameter) containing 10 ml distilled water, and then placed in an incubator at 37°C. The WVTR was calculated using the following equation:
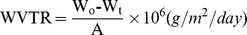
Where WVTR is expressed in g/m^2^/day, A (mm^2^) is the bottle mouth area, W_o_ (g) and W_f_ (g) are the weights of the device before and after being placed in an incubator for 24 h, respectively. The results are presented as a mean value with a standard deviation (n = 6).

The in vitro degradation experiments were carried out in PBS at 37°C. The dry films were weighed and immersed in PBS for different time intervals during 70 days. Then the samples were freeze-dried for 24 h to remove excess water, and weighed again. Finally, the remainder weight was weighed to evaluate the stability of pure CS and CS/KGM blend films using the following equation.
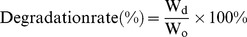
Where degradation rate is expressed in %, and W_o_ and W_d_ are the weights of the films before and after degradation for a specific time interval, respectively. The results are presented as a mean value with a standard deviation (n = 6).

### In vitro Gentamicin release from Poly (DEX-GMA/AAc) nanoparticles

Dynamic dialysis method was employed to determine in vitro drug release profile of Gentamincin loaded Poly (DEX-GMA/AAc) nanoparticles. The drug content in the drug-loaded nanoparticles was determined upon natural degradation of nanoparticles without enzyme and drug release caused by nanoparticles swelling due to AAc residue on the polymer backbone. An amount exactly weight (0.5 g) of drug-loaded nanoparticles dispersed in PBS (3 mL) was introduced into dialysis bag and dialysis against PBS (50 mL). Dialysate was incubated at 37°C under magnetically stirring. At each time interval, Dialysate (1.5 mL) was taken out and carefully transferred to a test tube. After each measurement, 1.5 mL of fresh buffer was added. After each measurement, 3 mL of fresh buffer was added. 5 mL derivatization regent was added into each sample and 310 nm was set as detective wavelength.

### In vivo wound healing experiments

The wounds below were created in animals to mimic the conditions encountered in the surgical patients. The animal experiments were approved by the Ethics Committee of the Fourth Military Medical University. Rats were randomly divided into 5 groups and anesthetized with ethyl ether. A1.5 cm-wide wound was cut with scissors on the back of each shaved rat down to the fascia layer. Rats of 4 groups were dressed with pure CS and CS/KGM blend films and the lasted group was dressed with gauze as control. Wound closure observation was assessed by digital camera in the day 3, 7 and 14. The wound closure rate was calculated using the following equation:
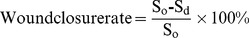
Where the wound closure rate is expressed as a percentage %, and S_o_ and S_d_ are the area of the originally created wound, and the area of the wound post-operative for a specific time interval, respectively. The results are presented as a mean value with a standard deviation (n = 6).

In addition, the wounds and the surrounding skin of post-operative for day 7 and 14 were fixed with 4% formaldehyde solution, paraffin embedded and stained with hematoxylin-eosin (HE) reagent for histological examinations.

### Preparation of GNPs-CS/KGM

GNPs-CS/KGM was prepared according to the procedure of C75K25 film with some modification. After purification of KGM, CS solution (60 mL) was mixed with KGM solution (20 mL) and 0.2 g Gentamicin loaded nanoparticles was added into the mixture to form GNPs-CS/KGM.

### In vitro Gentamicin release from GNPs-CS/KGM

Dynamic dialysis method as described above was also employed to determine in vitro drug release profile of Gentamincin from GNPs-CS/KGM. An amount exactly weight (1.0 g) of drug-loaded GNPs-CS/KGM in PBS (3 mL) was introduced into dialysis bag and dialysis against PBS (50 mL). Dialysate was incubated at RT under magnetically stirring. At each time interval in 72 h, Dialysate (3 mL) was taken out and carefully transferred to a test tube. After each measurement, 3 mL of fresh buffer was added. 5 mL derivatization regent was added into each sample and 310 nm was set as detective wavelength.

### Biocompatibility evaluation of GNPs-CS/KGM

Mice skin fibroblast L929, obtained from stomatological hospital affiliated with Fourth military medical University, was used to assay the cytotoxicity on the Poly (dex-GMA/AAc) nanoparticles, C75K25 film and blank NPs-CS/KGM. Poly (dex-GMA/AAc) nanoparticles, C75K25 film and blank NPs-CS/KGM were added into DMEM cell culture medium with 10% FBS to obtain mixture of 0.1 g/mL, and extractedfor 24 h in incubator at 37°C. After applying extracts, medium was suck out and diluted using fresh DMEM medium with 10% FBS to make different concentration gradient sample solution (0.1, 0.05 and 0.01 g/mL). L929 cell suspension (1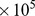/mL, 100 µL/well) was seeded in 96 well plates, and cultured in a humidified incubator at 37°C with 95% air and 5% CO_2_ for 24 h. Sample solution of different concentration (100 µL/well) was added separately and cultured for another 1d, 3d, and 5d. The medium was suck out and MTT (0.5 mg/ml, 20 µL/well) with medium (100 µL/well) was added in each well, then culturedfor 4 h. The medium was then discarded. Dimethylsulfoxide (DMSO) (150 µL) was added into each well and OD value of the solutions was measured at 490 nm using plate reader (xMark). Mean values were obtained from six wells per group.

### Antibacterial activity evaluation

Antimicrobial activity tests of Poly (dex-GMA/AAc) nanoparticles, C75K25 film and GNPs-CS/KGM were carried out using agar diffusion method. C75K25 film and GNPs-CS/KGM were cut into a disc form of 7 mm diameter using a puncher and nanoparticles were scattered on sheep blood agar plates, which had been previously seeded with inoculum (0.1 ml, 0.5 McIntosh concentrations) of Staphylococcus aureus ATCC 25923, Escherichia coli ATCC 25922, and Green copper pseudomonas ATCC 27853. The plates were then incubated at 37°C for 16 h. The diameters of inhibitory zones surrounding film discs as well as the contact areas of edible films with agar surface were then measured (n = 6).

### In vivo wound healing experiments

This study was conducted in accordance with Guide for the Care and Use of Laboratory Animals. Male Sprague-Dawley rats (250–400 g) were used for the study. They were anesthetized with an intraperitoneal injection of pentobarbital (50 mg/kg, Jinan Haohua Industry Co., Ltd). The rat's femoral vein was exposed and cut with scissors. Poly (dex-GMA/AAc) nanoparticles, C75K25 film, native CS film and GNPs-CS/KGM were applied to the area of hemorrhage, separately, and firm pressure was applied. In order to ensure that the dressings was tightly adhered to wound area, analytical weights of 50 g was used on the dressing to give a certain pressure. At the meanwhile, timers was started and ended until bleeding was stopped. Spilled blood was suck up using gauze after weighing, and blood loss was calculated by gauze and sample mass difference before and after bleeding. Yunnan baiyao powder was chosen as positive control and gauze to cover hemostatic as negative control. Hemostatic performance was evaluated by blood loss and hemostatic time. (n = 6).

Rats were randomly divided into 2 groups and anesthetized with ethyl ether. A1.5 cm-wide wound was cut with scissors on the back of each shaved rat down to the fascia layer. One group was dressed with GNPs-CS/KGM and the other group was dressed with gauze as control. Wound closure observation was assessed by digital camera in the day 3, 7 and 14. The wound closure rate was calculated using the same equation as mentioned above.

In addition, the wounds and the surrounding skin of post-operative for day 7 and 14 were fixed with 4% formaldehyde solution, paraffin embedded and stained with hematoxylin-eosin (HE) reagent for histological examinations.

### Statistical analyses

Statistical analyses were performed with software (SPSS 13.0). Primary statistical analysis of data (mean ± standard deviations) was performed with the analysis of variance (ANOVA) to determine mean differences in every tested group, followed by Student-Newman-Keuls –q (SNK-q) test for multiple comparisons. P<0.05 was considered to indicate statistical significance.

### Ethical approval

The animal experiments were approved by the Ethics Committee of the Fourth Military Medical University.

## Results and Discussion

### Characterization of polymer and nanoparticles

**Figure 2:**
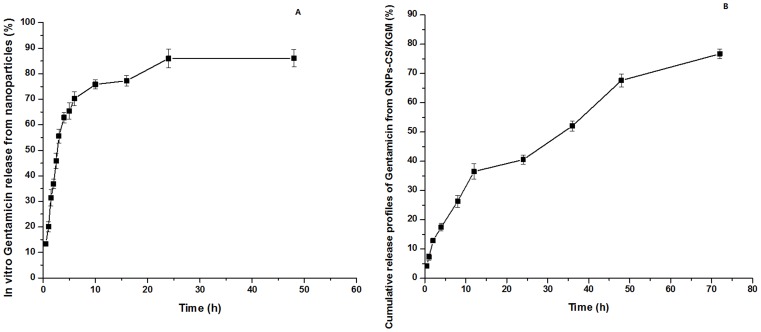
^1^H-NMR spectra of DEX-GMA. (a), Morphology of nanoparticles observed by TEM (b) (A: blank nanoparticles, B: drug loaded nanoparticles), Particle size distribution from DLS analysis (c) (A: blank nanoparticles, B: drug loaded nanoparticles).

^1^H-NMR spectra of DEX-GMA were recorded with a Bruker AC at 500 MHz with D_2_O as solvent. As shown in spectra (Fig. 2a), the signal from the proton at the anomeric carbon of the α-1, 6 linkages at 4.9 ppm) was well separated from the multiplet peaks of from 3.4 to 4.0 ppm. The typical peaks from the methacryloyl group were observed at 1.95 ppm (methyl protons) as well as at 5.9 and 6.2 ppm (protons at the double bond).

Poly (DEX-GMA/AAc) blank nanoparticles and Gentamicin loaded nanoparticles were all obtained by free radical emulsion polymerization. Encapsulation and loading efficiency were 89.92±1.47% and 7.15±0.16% calculated according to Gentamincin standard curve (A = 0.0227C-0.0408, R^2^ = 0.9996, linear range, 10–35μg/mL). The shape, morphology and size of nanoparticles obtained from Poly (DEX-GMA/AAc) blank nanoparticles and Gentamicin loaded nanoparticles were analyzed by TEM and DLS. The TEM image of freeze-dried nanoparticles was presented in Fig. 2b. The size distribution of nanoparticles in water was shown in Fig. 2c. It could be seen from DLS data that the blank and drug loaded nanoparticles diameters had peak at 50–100 nm, which was nearly consistent with the TEM and images.

**Figure 3:**
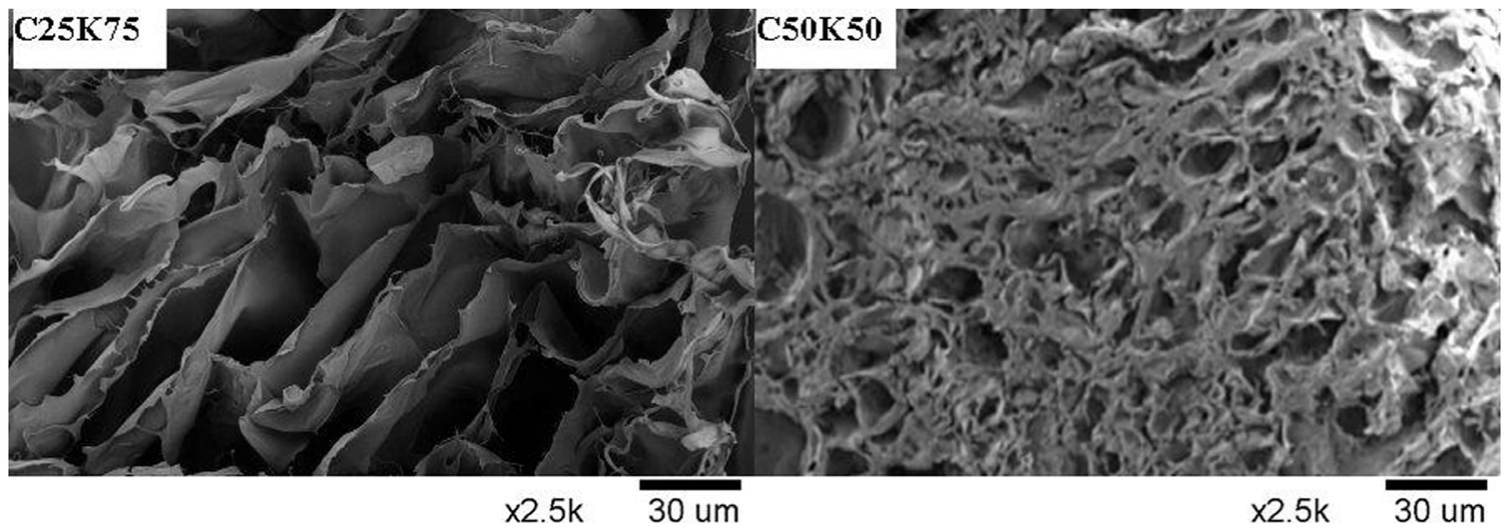
In vitro cumulative release profile of Gentamicin. From Poly (DEX-GMA/AAc) nanoparticles (A) and GNPs-CS/KGM (B).

In vitro cumulative release profiles of Gentamicin from nanoparticles are shown in Fig. 3a. The release profiles appeared to have two phases. The first phase was a rapid release in the prior period and about 70% were released in this phase (within the first 6 h). The rapid releasing process was mainly due to the nanoparticles surface drugs could easily diffuse in the initial time and the swelling of nanoparticles promoted drug release. The second phase was a relatively slow release ranging from 6 to 24 h, and about 80% drug were released in this phase due to the swelling equilibrium and degradation of nanoparticles. The dissolved drugs were diffused into the release medium. The cumulative percentage release of drug from nanoparticles was about 83% for 72 h.

As shown in Fig. 3b, Gentamicin release rate of GNPs-CS/KGM has sustainable increase in 36 h and then drug release became slower. The cumulative drug release rate was about 75% for 72 h.

### Characterization of KGM/CS blend film

**Figure 4:**
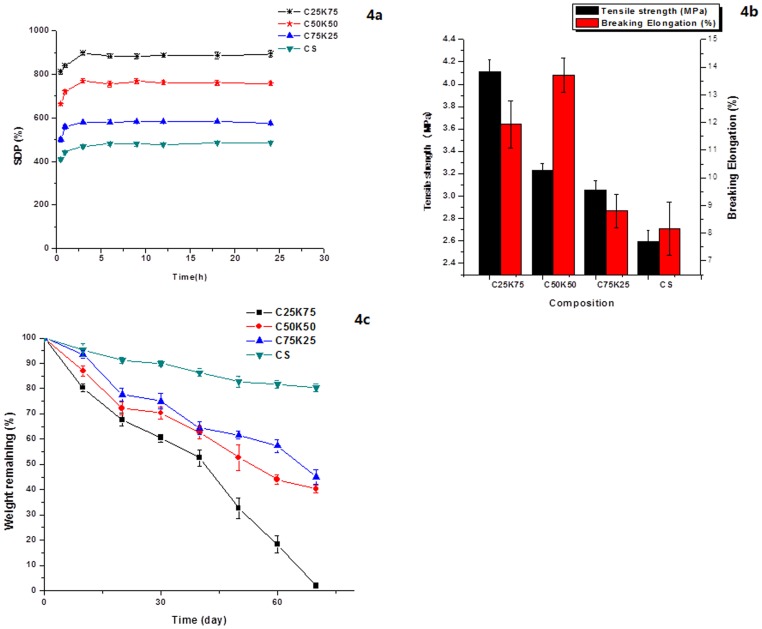
The cross-section morphology of blending film with different KGM/CS ratio by SEM.

Fig. 4 shows the cross-section morphology for KGM/CS blending film. The three-dimensional network structure of film was observed very clearly, which was similar to our previous work. The porous structure was benefit for cell adhesion and proliferation as well as promotes the growth of tissue. The porous layer was arranged very regularly, which was benefit to separate components through the membranes and improve of the flux. Porous C25K75 films had pores with an average diameter of 95±16 µm and C50K50 film of 10±7 µm. The pore size of CS and C75K25 film were between them. KGM has stronger swelling capacity which resulted in increased pore size while amino of CS could form hydrogen bonds with hydroxy of KGM. This duality influence lead to the result that the tendency of increase with the pore size appeared smaller before alonging with increasing percentage of KGM. So when KGM/CS proportion became 1∶1, the pore size reached smallest.

**Figure 5:**
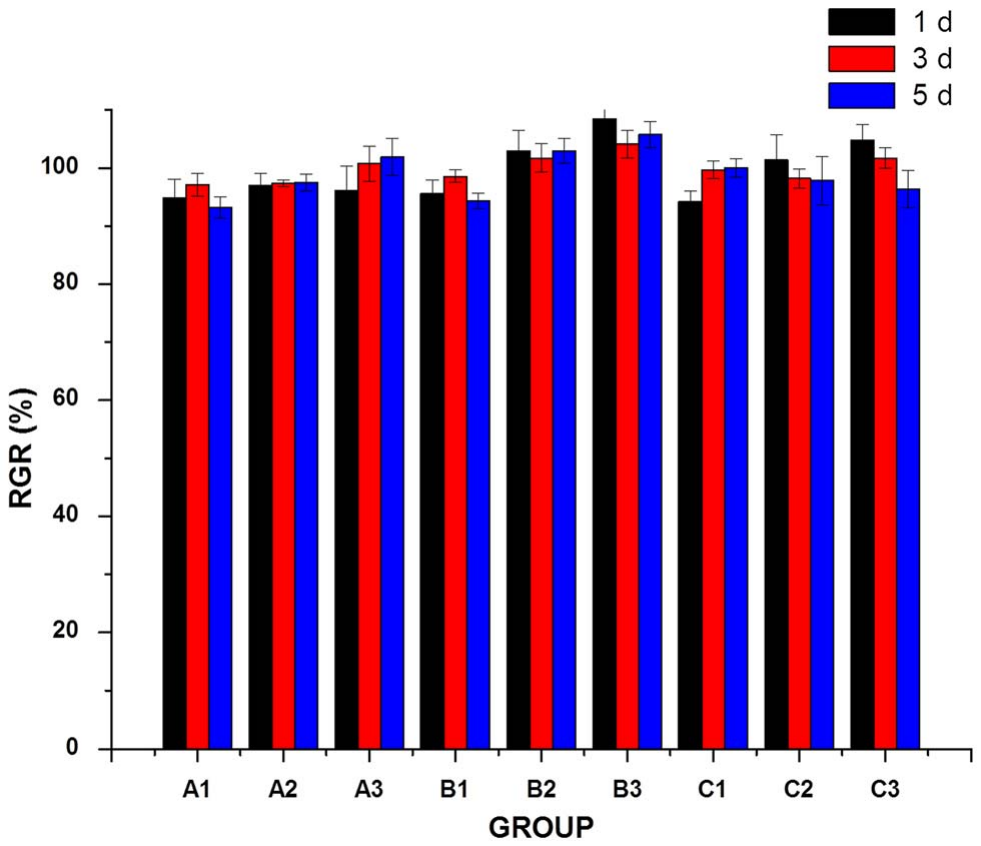
Degree of water uptake of the pure CS and KGM/CS blending films (a), TS and breaking elongation of KGM/CS blending films with different KGM/CS ratio (b), Degradation profile of KGM/CS blending films with different KGM/CS ratio (c).

To study the effects of KGM on blend film swelling, the degree of swelling was plotted with different KGM/CS ratio coded as K25C75, K50C50, and K75C25 for 72 h, as shown in Fig. 5a. It was noticed that all films has strong capability of water uptake in 3 h due to strong interaction between water molecules and the membrane containing OH groups and NH_2_ groups [37]. The degree of swelling of blend films increased with more KGM due to its good hydrophilicity. SDP of C25K75 reached (890±36) %. After 18 h, swelling rate of blend films decreased due to dissolution of KGM.

Water vapor transmission rate (WVTR) was one of the most important indicators to evaluate the WVTR moisturizing performance of wound dressing and hemostasis material. It was generally acknowledged that wound dressings with WVTR between 2000 to 5000 g·m^−2^·day^−1^ could prevent excess dehydration of wound surface, maintain wet environment of wound and avoid excess accumulation of exudate [38]. WVTR of films in this study was found to be between 2282 to 3170 g·m^−2^·day^−1^, for instance, WVTR of CS film was 2950 g·m^−2^·day^−1^ and C75K25 was 2282 g·m^−2^·day^−1^. As shown in Table 1, the WVTR of CS/KGM films showed better water uptake ability than that of CS itself due to good hydrophilcity of KGM and large pore size of CS itself. And the results of WVTR showed that blend films obtained in our study could prevent water evaporation effectively and provided a good moist environment for wound.

**Table 1:** The water vapor transmission rate of different composition of CS/KGM films.

Composition (CS/KGM) (wt%)	WVTR (g·m^−2^·day^−1^)
**C25K75**	3170 ± 36
**C50K50**	2580 ± 90
**C75K25**	2282 ± 73
**CS**	2950 ± 44

Mean ± S.D. (n = 6).

Mechanical strength is one of indicators to evaluate materials mechanical properties. As shown in Fig. 5b, TS of the films increased significantly from 2.6 MPa to 4 MPa with increased KGM composition and breaking elongation was about 10%. The results showed that mechanical strength of film was significantly enhanced by mixing KGM with CS. It was probably because hydrogen bond created by –NH2 protonation of CS and hydroxyl of KGM enhanced intermolecular forces and then increased tensile strength of film.

Degradation profiles of CS, C25K75, C50K50, and C75K25 films are shown in Fig. 5c. It was revealed by the results that the ratio of KGM has significantly influence on degradation rate of film. Degradation rate of blend films increased with increased KGM ratio. After 70 days, C25K75 film was almost completely degraded while residual mass of C50K50 and C75K25 film was about 40% and CS film was 80%.

### Biocompatibility evaluation of GNPs-CS/KGM

**Figure 6:**
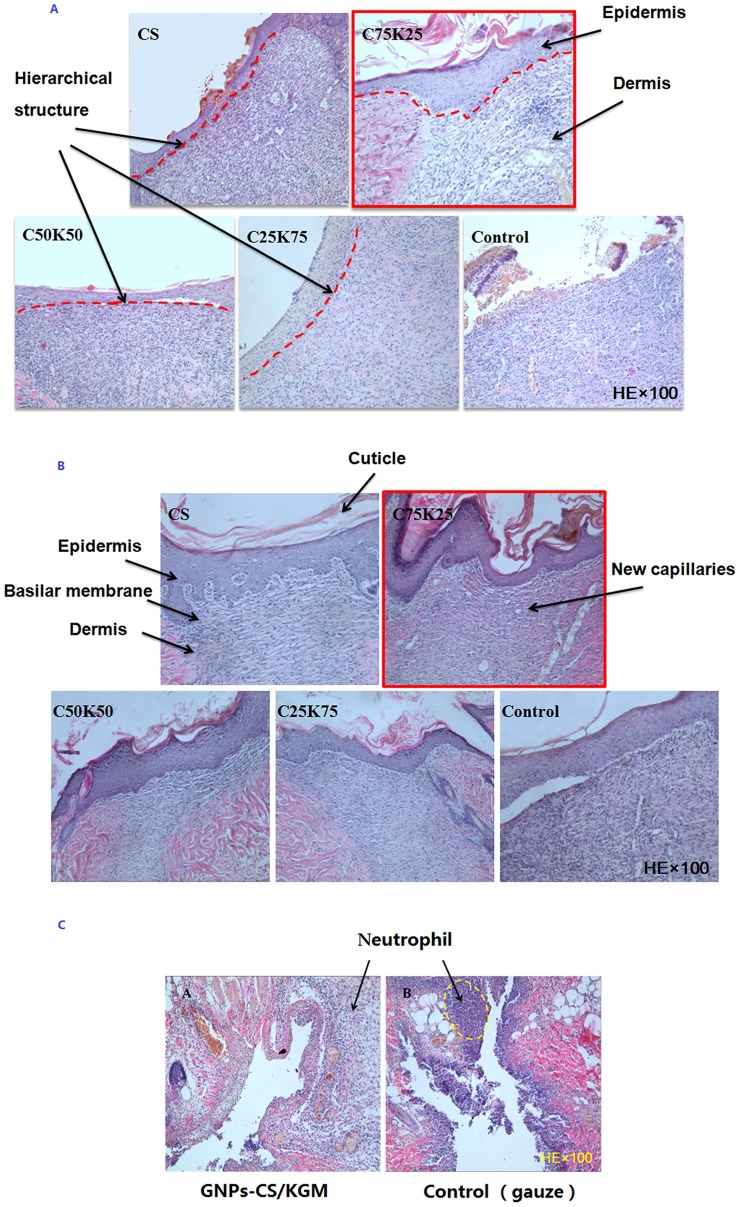
Histological examination by HE staining. Effect of different KGM/CS formulae, CS film and control group on incision wound in rat skin (A) after 7d, (B) after 14d, (C) GNPs-CS/KGM treated after 3d.

The MTT assay is an indirect method of assaying cell growth and proliferation since the A490 values can be correlated to the cell number. As the basis of cell growth, proliferation and differentiation, cell attachment is an important measure to evaluate the biocompatibility of biomaterials. To assess cellular adhesion, Poly (dex-GMA/AAc) nanoparticles, C75K25 film and blank NPs-CS/KGM were seeded with the same density of human fibroblasts. The cell viabilities measured by MTT assay of fibroblasts cultured on different materials were shown in Fig. 6. The OD value of each tested group had no obvious difference with control group. Cell relative growth rate of tested groups were higher than 90%, especially in C75K25 group with low concentration, relative growth rate appeared to be higher than 100%. The MTT results revealed that Poly (dex-GMA/AAc) nanoparticles, C75K25 film and blank NPs-CS/KGM had good biocompatibility and no cytotoxicity.

### Wound healing effects of KGM/CS blend films and GNPs-CS/KGM

Wound healing is an interaction of a complex cascade of biochemical and cellular events that generates resurfacing, reconstitution and restoration of the tensile strength of injured skin [39]. For evaluation of the wound healing capability of the preparations, percent wound contraction on incision wounds and histopathological studies were measured. First, we studied wound healing effects of blend film. As shown in Table 2, it was observed that postoperative wound area had slightly contraction after 3d. The cut began to scab after 1 week and the scab become detached after 2 weeks with significant wound shrinkage.

**Table 2:** Effect of KGM/CS films, CS film and control on wound area contraction during 14 days (values are mean ± S.D., n = 6 observations in each group).

Group	Percent wound contraction (%) Day 3		
	Day 3	Day 7	Day 14
**C25K75**	10. 4±4.21	28.4 ±5.34	79.1 ±4.26^*^
**C50K50**	15.2 ±3.26^*^	31.1±5.27^*^	80.7 ±5.69^**^
**C75K25**	18.9 ±4.18^**^	32.0±4.24^**^	81.1 ±3.76^**^
**CS**	16.8 ±5.21^*^	30.7 ±3.43^*^	80.4 ±4.48^**^
**Control (gauze)**	7.9 ±2.65	22.7 ±3.12	70.8 ±2.85

Values are significant (^*^) at P<0.05 and (^**^) at P<0.01as compared to the control group.

**Figure 7:**
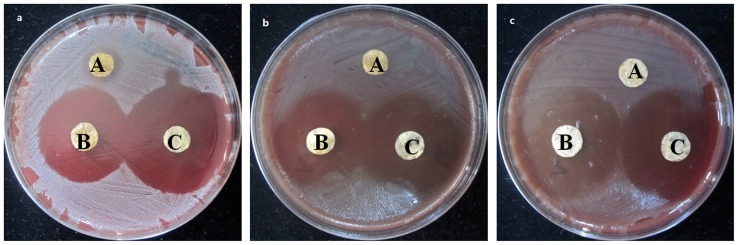
Inhibitory effect against the bacteria by Disc agar diffusion. A, B, and C presented C75K25 film, Drug loaded Poly (dex-GMA/AAc) nanoparticles and GNPs-CS/KGM treated with three kinds of bacteria, respectively. (a) Staphylococcus aureus ATCC25923, (b) Escherichia coli ATCC25922, (c) Green copper pseudomonas ATCC27853.

Healing of closed incisional wounds was also determined by the histopathological studies. Fig. 7 shows the histological studies on different KGM/CS formulae, CS film and gauze control group. Granulation tissue of wound became thickening gradually along with the increasing healing time. It was revealed by HE staining that inflammatory infiltration of different degree could be observed after 3d on all experiment and control group. Epidermal cell layer of treatment group proliferated actively after 7d and capillaries began to form in dermis. Hierarchical structure was visible between epidermis and dermis. Epithelial structure such as stratum basale and acanthosis cell layer was observed in CS and C75K25 treatment group obviously, especially in C75K25 group, cuticular layer was also apparently visible. However, in gauge control group, no clear dermal tissue structures were formed and there was no hierarchical structure between epidermis and dermis after 7d. After 14d, the photomicrographs for the section of incision wound treated with treatment group especially C75K25 group showed significant hierarchical structure of epithelial tissue covering the wound area together with remodeling of well-developed collagen fibers that almost resembled normal tissue while in control group, there was still actively proliferated fibroblast in dermis. Cuticle, stratum granulosum, spinous cells layer, and basalis stratified clearly. Sections obtained from incision wounds treated with C75K25 revealed almost complete healing with nearly full resolution of the granulation tissue, normal tissue architecture, and new capillary distribution. It was known that CS itself has promoting wound healing effect and mixed with KGM could improve its mechanical properties. However, if the amount of KGM increased in CS/KGM, the solubility of film will increase and film-forming property will decrease. So when the proportion between CS and KGM reached 75 to 25, the CS/KGM film has the best mechanical properties and film-forming property. That's why the histology study appears to favor the C75K25 films over the other formulations.

Then GNPs-CS/KGM was also studied for wound healing effects. As shown in Table 3, contraction rate of GNPs-CS/KGM has significantly increased compared with gauze group (*P*<0.05) indicating that GNPs-CS/KGM could effectively promote contractility of wound and has great potential as wound dressing materials. It was also revealed by HE staining that inflammatory infiltration of GNPs-CS/KGM treatment group were apparent less than control group (Fig. 7).

**Table 3:** Effect of GNPs-CS/KGM and control on wound area contraction during 14 days (values are mean ± S.D., n = 6 observations in each group).

Group	Percent wound contraction (%) Day 3		
	Day 3	Day 7	Day 14
**GNPs-CS/KGM**	19.7 ±2.28^**^	33.4±3.87^**^	82.8 ±4.25^**^
**Control (gauze)**	7.6±2.92	24.2±3.46	72.9 ±3.45

Values are significant (^*^) at P<0.05 and (^**^) at P<0.01as compared to the control group.

### Antibacterial activity evaluation

**Figure 8:**
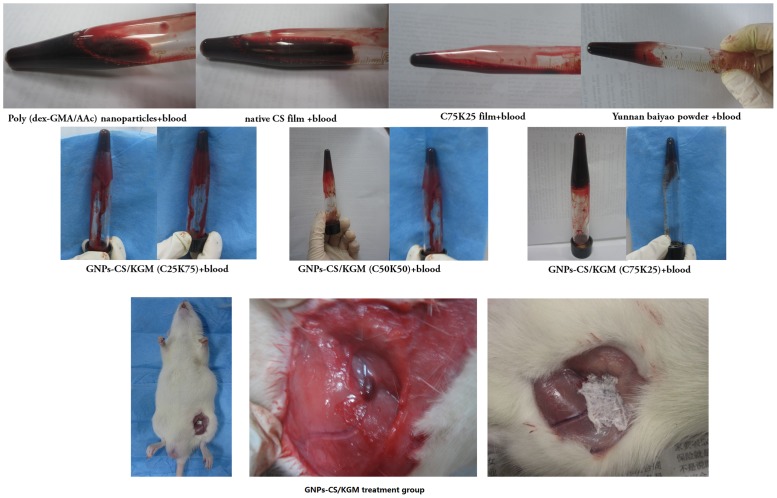
Effect of 0.25 wt% samples on heparinized rats blood and rat's femoral vein injury treated with GNPs-CS/KGM group.

Drug loaded Poly (dex-GMA/AAc) nanoparticles, C75K25 film and GNPs-CS/KGM were prepared in vitro for their microbial activity against Staphylococcus aureus, Escherichia coli, and Green copper pseudomonas using disc diffusion method. It was revealed by Fig. 8 that GNPs-CS/KGM and Drug loaded Poly (dex-GMA/AAc) nanoparticles had strong inhibitory effect against the bacteria mentioned above while C75K25 film only had inhibitory effect against Staphylococcus aureus. The diameter of Bacteriostatic ring was shown in Table 4.

**Table 4:** The Bacteriostatic ring diameter of C75K25 film, Drug loaded Poly (dex-GMA/AAc) nanoparticles and GNPs-CS/KGM against different bacterial strain (values are mean ± S.D., n = 6 observations in each group).

Group	Staphylococcus aureus	Escherichia coli	Green copper pseudomonas
**C75K25 film**	8.6±0.73	N	N
**GNPs-CS/KGM**	21.3±0.79	21.5±0.70	22.8±0.51
**Drug loaded Poly (dex-GMA/AAc) nanoparticles**	25.1±0.82	22.3±0.74	22.4±0.89

### Hemostatic activities evaluation

First, we compared the effects of Poly (dex-GMA/AAc) nanoparticles, C75K25 film, GNPs-CS/KGM, native CS film and Yunnan baiyao powder as positive control on heparinized rat whole blood. In each case, we adjusted the polymer concentration in the overall mixture to 0.25 wt%. Upon addition 3 kinds of GNPs-CS/KGM, liquid blood is instantly transformed into a self-supporting gel, as can be seen from the photograph in Fig. 8 where the sample holds its weight upon tube inversion. In comparison, the rest samples and blood remains a freely flowing liquid, as seen from its corresponding photograph.

According to the result above, we studied hemostatic time and bleeding volume of different treatment group. As shown in Table 5, GNPs-CS/KGM and Yunnan baiyao powder group has effectively shorten hemostatic time and significantly decreased bleeding volume compared with control group (*P*<0.05). The results indicated that the hemostatic effect of GNPs-CS/KGM and Yunnan baiyao powder was superior to gauze and GNPs-CS/KGM has stronger hemostatic effect.

**Table 5:** Hemostatic time and bleeding volume of different treatment group (values are mean ± S.D., n = 6 observations in each group).

Group	hemostatic time (s)	bleeding volume (g/kg)
**Poly (dex-GMA/AAc) nanoparticles**	256±18^*^	2.10±0.32^*^
**C75K25 film**	201±17^**^	1.40±0.10^**^
**GNPs-CS/KGM**	176±14^**^	0.95±0.20^**^
**native CS film**	249±9^*^	2.38±0.48^*^
**Yunnan baiyao (positive control)**	207±12^**^	1.45±0.22^**^
**Control (gauze)**	360±26	2.42±0.47

Values are significant (^*^) at P<0.05 and (^**^) at P<0.01as compared to the control group.

### Conclusion

In this study, GNPs-CS/KGM was prepared as novel wound dressing with hemostatic capabilities and antibiotic effect. The result revealed that microporous structure of CS/KGM has the ability of effectively haemostasis. GNP could one step further promote blood clotting due to its physicochemical properties. GNPs-CS/KGM has the ability to transform whole liquid blood into a gel, and it quickly stops bleeding from injuries in small animals. An additional important aspect is GNPs-CS/KGM has significant wound healing effect and hemostatic properties. Besides, GNPs-CS/KGM obtained good antibiotics ability by addition of gentamicin in NPs. Thus, GNPs-CS/KGM may be considered promising biodegradable and biocompatible novel wound dressing with hemostatic capabilities as well as antibiotic effect for treatment of external bleeding injuries.
